# Artificial intelligence in respiratory pandemics—ready for disease X? A scoping review

**DOI:** 10.1007/s00330-024-11183-8

**Published:** 2024-11-21

**Authors:** Jennifer Straub, Enrique Estrada Lobato, Diana Paez, Georg Langs, Helmut Prosch

**Affiliations:** 1 Computational Imaging Research Lab, Department of Biomedical Imaging and Image-guided Therapy, Medical University of Vienna, 1090 Vienna, Austria; 2 Nuclear Medicine and Diagnostic Imaging Section, Division of Human Health, Department of Nuclear Sciences and Applications, International Atomic Energy Agency (IAEA), 1220 Vienna, Austria; 3 Christian Doppler Laboratory for Machine Learning Driven Precision Imaging, Department of Biomedical Imaging and Image-guided Therapy, Medical University of Vienna, 1090 Vienna, Austria; 4 Division of General and Paediatric Radiology, Department of Biomedical Imaging and Image-guided Therapy, Medical University of Vienna, 1090 Vienna, Austria

**Keywords:** Radiology, Respiratory pandemic, Data collection, Artificial intelligence

## Abstract

**Objectives:**

This study aims to identify repeated previous shortcomings in medical imaging data collection, curation, and AI-based analysis during the early phase of respiratory pandemics. Based on the results, it seeks to highlight essential steps for improving future pandemic preparedness.

**Materials and methods:**

We searched PubMed/MEDLINE, Scopus, and Cochrane Reviews for articles published from January 1, 2000, to December 31, 2021, using the terms “imaging” or “radiology” or “radiography” or “CT” or “x-ray” combined with “SARS,” “MERS,” “H1N1,” or “COVID-19.” WHO and CDC Databases were searched for case definitions.

**Results:**

Over the last 20 years, the world faced several international health emergencies caused by respiratory diseases such as SARS, MERS, H1N1, and COVID-19. During the same period, major technological advances enabled the analysis of vast amounts of imaging data and the continual development of artificial intelligence algorithms to support radiological diagnosis and prognosis. Timely availability of data proved critical, but so far, data collection attempts were initialized only as individual responses to each outbreak, leading to long delays and hampering unified guidelines and data-driven technology to support the management of pandemic outbreaks. Our findings highlight the multifaceted role of imaging in the early stages of SARS, MERS, H1N1, and COVID-19, and outline possible actions for advancing future pandemic preparedness.

**Conclusions:**

Advancing international cooperation and action on these topics is essential to create a functional, effective, and rapid counteraction system to future respiratory pandemics exploiting state of the art imaging and artificial intelligence.

**Key Points:**

***Question*** *What has been the role of radiological data for diagnosis and prognosis in early respiratory pandemics and what challenges were present?*

***Findings*** *International cooperation is essential to developing an effective rapid response system for future respiratory pandemics using advanced imaging and artificial intelligence.*

***Clinical relevance*** *Strengthening global collaboration and leveraging cutting-edge imaging and artificial intelligence are crucial for developing rapid and effective response systems. This approach is essential for improving patient outcomes and managing future respiratory pandemics more effectively.*

**Graphical Abstract:**

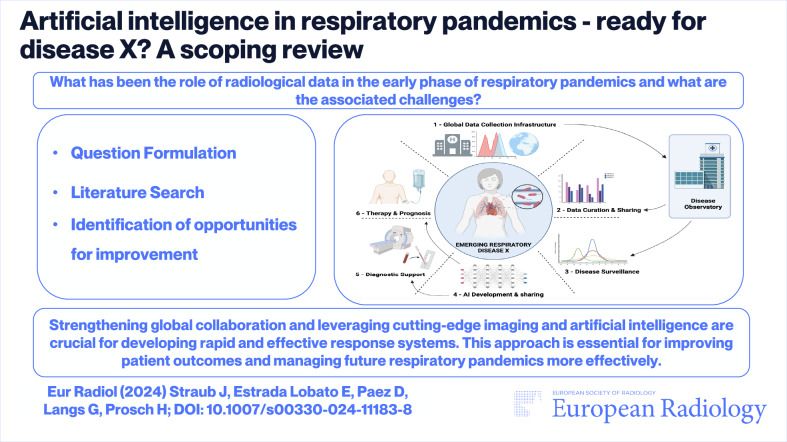

## Introduction

Over the past two decades, multiple outbreaks of respiratory diseases caused so-called Public Health Emergencies of International Concern (PHEIC) [1], including SARS in 2002, H1N1 influenza in 2009, MERS since 2012, and COVID-19 since 2019. The World Health Organization (WHO) is responsible for declaring a pandemic and monitors disease activity globally through a network of centers. It has created a pandemic preparedness plan, and established so-called “priority diseases,” including the abovementioned viruses [2, 3].

Medical imaging plays an important role in the diagnosis, prognosis, follow-up, and therapy guidance of respiratory diseases [4–7]. Especially in early pandemic phases, radiological data collection, curation and distribution are essential for an optimized response to a previously unknown pathogen, which has only recently been exemplified through COVID-19 [8, 9]. Further, artificial intelligence (AI) has seen rapid development and increasing impact in the field of chest imaging over the past two decades. It has the potential to help detect outbreaks, forecast case numbers, guide drug development and optimize diagnosis and prognosis [10]. However, if data used for AI training, testing, and validation suffers from a lack of diversity, poorly defined diagnosis, or unknown confounding factors, its applicability is greatly hindered [11, 12]. Consequently, the frequency of outbreaks and their global impact on health systems raises questions about technical and procedural preparedness for respiratory diseases that are certain to emerge in the near future.

In this review, we assess the role of imaging in 21st-century respiratory pandemics and derive possible future applications of imaging and AI before and during the early stages of respiratory pandemics. We address the previous shortcomings and future requirements of international data compilation and distribution and outline actions to enable rapid and high-quality AI algorithm development to support the management of future respiratory disease outbreaks.

### Search strategy and selection criteria

We initially defined the following research questions:

- What was the role of radiology in the early phase of the pandemic in terms of diagnosis, therapy guidance, and prognosis?
- What efforts were made regarding (international) data collection?
- How was AI, in combination with thoracic imaging, used in early pandemics?

**Fig. 1:**
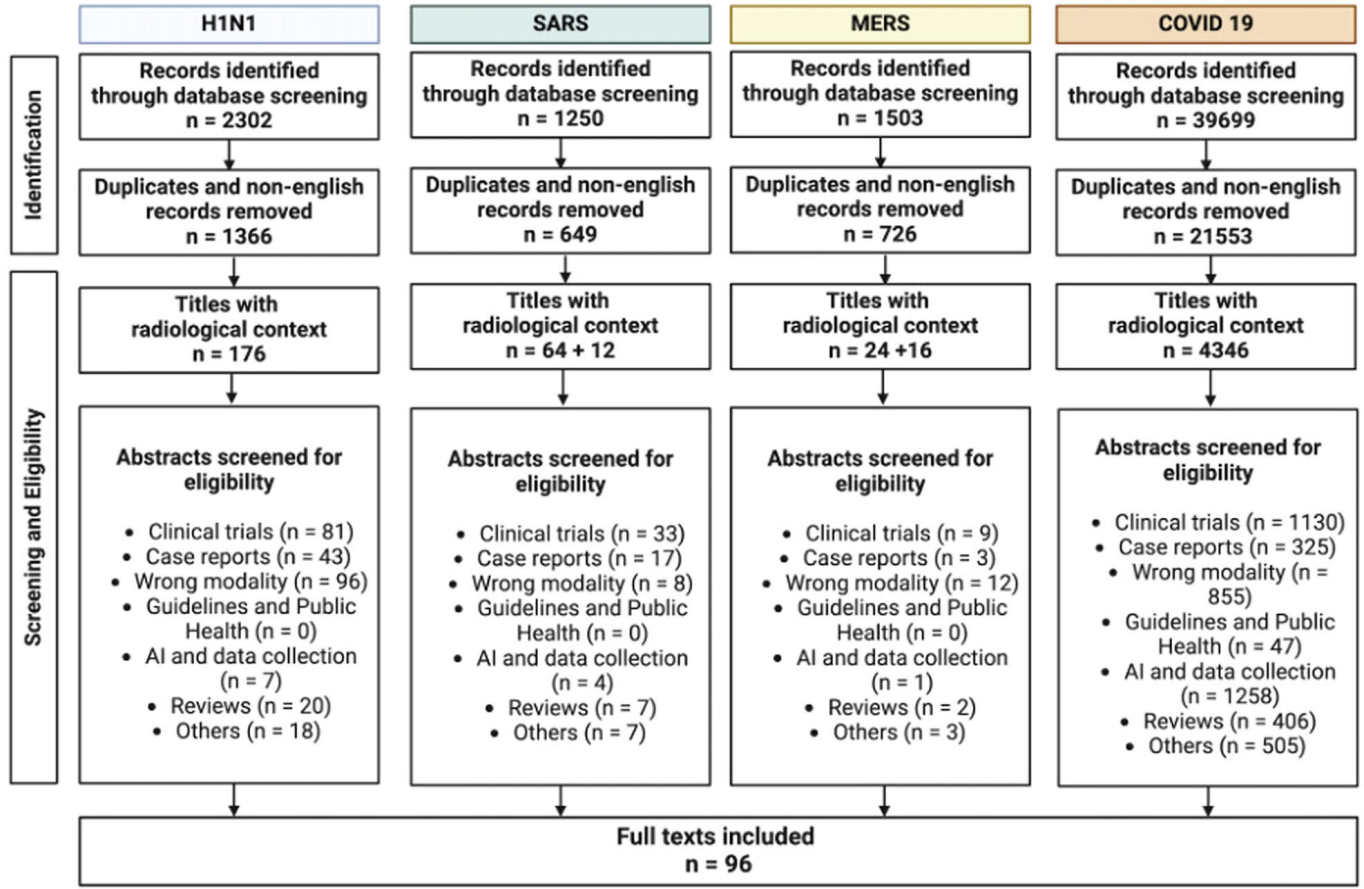
Screened literature for the scoping review

We searched PubMed/MEDLINE, Scopus, and Cochrane Reviews for articles published from January 1, 2000, to December 31, 2021, using Medical Subject Heading terms “imaging” or “radiology” or “radiography” or “CT” or “x-ray” in combination with each of the four pandemics, i.e., “SARS,” “MERS,” “H1N1” or “COVID-19.” Only articles in English were included. Articles within these searches were reviewed and analyzed for relevant cited references (cf. Fig. 1). Case definitions were identified through searches of the WHO and CDC institutional repositories. Studies and gray literature of any design, language, region, or timeframe, including commentaries, abstracts, and reviews, were considered eligible for inclusion.

We initially screened titles and abstracts, followed by reviewing of full texts and additional search of reference lists for related publications. Articles were extracted using a standardized form containing characteristics including authors, titles, dates and journals, study type. In accordance with the recommendations by Levac et al [13], we first analyzed the data, followed by a reporting of results and a final application of meaning to the results (cf. Fig. 1).

**Fig. 2:**
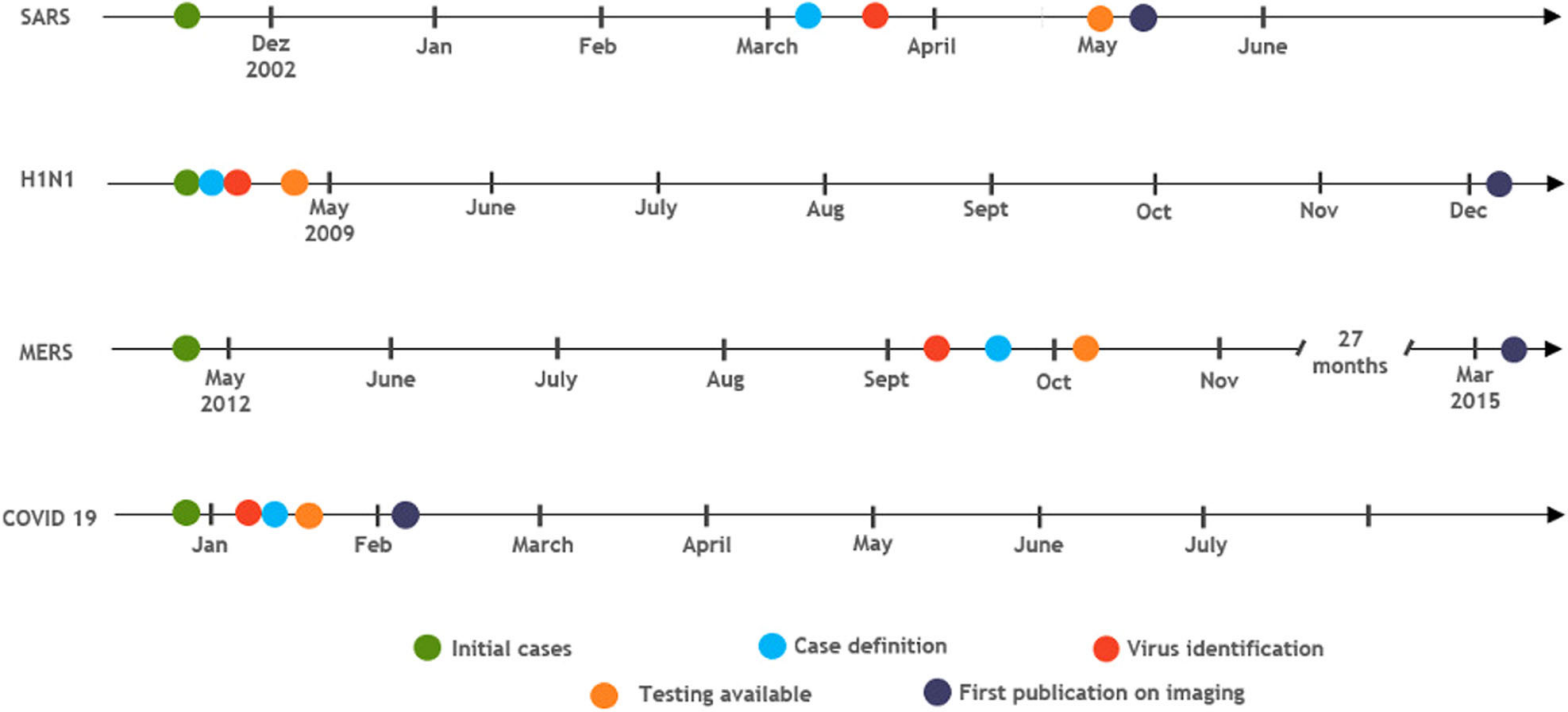
Key points in pandemic timelines. While the time from identification of a new pathogen to full genome sequencing and development of PCR-testing kits shortened to a few days over the last two decades, the time from infection of an initial case to identification of a new pathogen can remain comparably long, especially if cases occur sporadically. The swift identification of H1N1 was due to one of the first patients being randomly included in a clinical trial to evaluate an experimental diagnostic test [96], which further enhances the importance of routine surveillance in early pandemics. Time to publication of papers with a main focus on imaging varied greatly between outbreaks because research on this topic depends on the initiatives of individual authors and institutions

For each of the four diseases, more detailed timelines of published imaging findings, and the role of imaging in diagnosis, therapy guidance, and prognosis can be seen in Fig. 2.

## SARS

### Identification of a new pathogen and first imaging findings

On February 11, 2003, about 300 cases and five deaths of acute respiratory distress syndrome were reported by the WHO from the province of Guangdong in China. A month later, a global alert as well as a first case definition for this newly emerging disease were published. At that point, it had already spread to North America and was described as severe acute respiratory syndrome (SARS) [14, 15]. By March 21, 2003, the CDC published preliminary clinical and radiological descriptions. By March 24th, a novel coronavirus was identified as the causative agent, and its ribonucleic acid was sequenced within 3 weeks [16]. The first pandemic of the century was declared over on July 5, 2003, after spreading to 29 countries, infecting 8096 people and causing 774 deaths [14, 17].

### Role of imaging in the diagnosis, therapy guidance, and prognosis

An early local case definition from Guangdong province, as well as the first WHO case definition from March 15th, included chest radiographic changes making it a cornerstone of diagnosis and triage [18, 19]. Chest radiographic abnormalities were reported to precede respiratory symptoms in some cases [20–22]. Radiographs were regularly used to monitor therapy response in treatment trials [23], they formed the basis for treatment choices [24], and discharge was associated with improvement of radiographic findings following WHO guidelines. While radiographic follow-up was additionally recommended, little has been published on this subject [25, 26]. By the end of the pandemic no standard therapy existed, not least because many studies had not yet been published at that time [27].

### Data sharing, availability and AI applications

To increase pandemic preparedness, the International Health Regulations (IHR) were updated [28], and efforts were made for future standardized international data collection. However, they were hampered by legal reservations regarding publication rights and data sharing [29].

By June 2004, a first publication on automated detection of SARS on chest radiographs was published. Data from 59 patients was collected from multiple centers in Asia; however, the authors did not describe the developed algorithm in sufficient detail [30]. A second paper from 2005 was identified, reporting an approach using neural networks to differentiate SARS from pneumonia cases [31].

## H1N1

### Identification of a new pathogen and first imaging findings

On April 17, 2009, the Mexican General Directorate of Epidemiology (DGE) issued an epidemiological alert due to rising numbers of influenza-like respiratory illnesses. Two similar cases in California, USA, were reported by the CDC on April 21, 2009, most probably caused by human-to-human transmission [32]. Only a week later, the genomic sequence of a novel influenza A H1N1 virus was published by the WHO, and primers for PCR were distributed globally. During the early phase in March and April, there were 1918 suspected and 97 confirmed cases, including 84 deaths [33]. Early case reports by the CDC from April 30, 2009, included radiologically confirmed pneumonia for 15 out of 16 patients after hospital admission without providing detailed descriptions or images [33]. The IHR were activated on June 11, 2009, when the WHO declared the outbreak a phase six pandemic [34]. It was officially declared over in August 2010, after 18,500 laboratory-confirmed deaths from H1N1 infections [32, 35].

### Role of imaging in the diagnosis, therapy guidance, and prognosis

Diagnostic tests were available swiftly, and consequently, neither the first WHO, CDC nor DGE case definition included radiology, although reagents were scarce and tests showed a lack of sensitivity [36–38]. By the end of June 2009, a few of the first H1N1 radiographs and CT images were made available online by Mexican scientists with the aim of simplifying diagnosis [39]. While no studies with sufficient detail on the association of radiographic findings were available in the early stages of the outbreak [40], and radiographs and CT scans were not assessed for their diagnostic or prognostic potential by specific studies until December that year [5, 41]. Research on radiology to differentiate between H1N1 and other disease causes was called for early on, and if no diagnostic tests were available, a combination of high suspicion and radiographic findings was seen as sufficient to initiate antiviral therapy [40, 42]. In October 2009, a study showed that pneumonic infiltrates were associated with a higher probability of ICU admission [40]. As opposed to pharmacological guidelines, no radiological guidelines existed [43].

### Data sharing, availability and AI

The CDC developed data collection forms by April 28, 2009, requesting images of radiographs, CTs, MRIs, their respective acquisition dates, and reports, among other things. Despite this effort, no information on imaging had been shared when an extensive analysis of the collected records was issued in November [40, 44]. Similarly, the WHO requested clinical and laboratory data from the first 100 cases in each country via forms including questions about radiographic findings, but the results were highly heterogeneous and largely not useful for further analysis [45, 46].

Two years after the outbreak a paper on computer aided detection of H1N1 from CT scans was published, using texture analysis and support vector machines [47]. It was not until the outbreak of Covid-19 that H1N1 was automatically differentiated from other infectious diseases based on imaging [48, 49].

## MERS

### Identification of a new pathogen and first imaging findings

On June 13, 2012, the first case of Middle East respiratory syndrome (MERS) occurred in Jeddah, Saudi Arabia, and the discovery of a novel virus was mentioned by ProMED on Sept 15, 2012 [50, 51]. Three days later, another infection with the same coronavirus was reported by the WHO [52]. A first case definition was published on September 25, 2012, and by the beginning of October, a first case report was published, the viral genome was sequenced, and two RT-PCR assays were developed. On October 4, 2012, the first imaging results of a MERS patient were described as bilateral basal consolidations as part of a case report [51]. Clusters, sporadic, and nosocomial infections keep occurring infrequently until today, but secondary infections are uncommon and require close contact with an infected individual. By October 2021, the WHO reported 2578 laboratory-confirmed cases and 888 deaths in 27 countries and declared MERS a priority pathogen [53].

### Role of imaging in the diagnosis, therapy guidance, and prognosis

As a consequence of low prevalence and almost instantly available PCR tests, radiology was no key component of the case definition [54]. When cases dramatically increased in Jeddah in spring 2014, the WHO case definition was adapted to prevalence changes. Patients with radiological signs of pneumonia were classified as suspected cases and PCR-tested [6]. As of 2022, no guidelines for the use of radiology for MERS exist. Outcome prediction using CTs and radiographs was analyzed in two retrospective studies in 2015 based on scores developed during the SARS pandemic. These studies showed an association of higher scores, pleural effusions, and the presence of comorbidities with increased mortality [6, 55–57].

### Data sharing, availability and AI applications

A need for structured clinical investigations including radiographs was noted by the WHO in March 2013 and announced the development of data collection forms as a countermeasure [58, 59]. In October, data sharing as regulated by the IHR was still an exception, due to insufficient resources, missing guidelines on how to conduct the required reports in practice, and a lack of legal basis [60]. The development of structured clinical trial forms by the WHO in 2018, 5 years after initially pointing out data gaps [61, 62], again underlined the lack of clearly coordinated response to this priority pathogen.

Further, not a single publication on automated image analysis in MERS cases was identified.

## COVID-19

### Identification of a new pathogen and first imaging findings

On December 31st, 2019, an outbreak of acute respiratory illness was reported to the WHO from China [63]. A week later, a coronavirus was identified as the causative agent, and its genome was sequenced by January 12th [64, 65]. Radiological findings were briefly described to enable or facilitate triage. Diagnosis was largely based on RT-PCR in the earliest days of the emerging pandemic when the number of suspected cases allowed comprehensive testing [66]. By the end of January, the outbreak expanded into a public health emergency of international concern, and on March 11th, it was declared a pandemic with more than 118,000 cases in 113 countries [67]. WHO clinical management guidelines from January 12, 2020, gave a brief initial description of imaging findings [66].

### Role of imaging in the diagnosis, therapy guidance, and prognosis

PCR tests were the primary diagnostic tool in mid-January [65, 66, 68], while CT scans showed higher sensitivity based on an analysis from early February [69]. Thoracic CTs formed the cornerstone of diagnosis in China due to insufficient test availability, and they were used for triage in northern Italy while waiting for PCR results [70]. Excessive case numbers and a lack of testing and CT capacities made it necessary to switch to x-ray in Brazil [71]. The International Society of Radiology reported significant differences between national guidelines [72], and by early April, the Fleischner Society was the first to provide resource-adapted guidance for radiology [73]. WHO guidance was published in June 11, 2020 [74]. Neither the initial WHO guidelines for clinical management nor their first situation report provided early advice on radiology in therapy, prognosis or follow-up [64, 66]. However, by the end of February, the probability of developing severe symptoms was shown to be associated with the extent of CT findings on admission [75], and a multicenter study showed links between abnormalities on admission and fatal outcome [76]. In April, a statement by the Fleischner Society as well as the ESR recommended restricting repeated radiographs to ICU patients with persisting poor or deteriorating status [73, 77]. The WHO recommended against the use of chest imaging for discharge decisions, while Chinese hospitals relied on it, and guidelines did not provide consistent advice [67, 72, 74].

### Data sharing, availability and AI

Data sharing was initialized by the WHO as of January 21, 2020, via a minimum data set report form [78]. It was created to collect clinical, demographical, and travel information, and merely included a checkbox for the presence of chest radiograph findings. Questions on radiology were completely removed from the second version of the revised form at the end of February [79].

At that time, two public datasets with less than 100 radiographs built the foundation for AI algorithm development aside from single-center approaches. As for CT, datasets with close to 2000 scans were used [80].

An initial high-quality data set was created by the RSNA by December 2020 under the name RICORD, which contained 240 CTs. It was, among numerous other national or institutional initiatives, followed by the 10.735 CT strong multicenter STOIC data set published in July 2021. The latter also contains valuable additional information on comorbidities, demographics, outcomes, and PCR results [81, 82].

A large number of artificial intelligence algorithms were developed during the early stage of the pandemic to aid diagnosis, but none were sufficiently reliable to be used without reservation. The reasons were studied extensively, and the primary reason was a lack of high-quality data availability [12]. As data became available, algorithm reliability improved [82, 83]. In some cases, such as the AI-based RadVid-19 project in Brazil, imaging and AI enabled rapid assessment of CT scans in hospitals without specialized radiologists [71].

## Lessons from previous outbreaks and the road ahead

In all outbreaks, the following pattern of radiology, prevalence, and resources emerged: Before the availability of testing (e.g., SARS 2002) diagnosis was based on contact history and radiological evidence [18, 19, 23]. When testing capacity was insufficient (e.g., first waves of COVID-19), CT scans and radiographs were used to accelerate diagnosis in triage settings in China and Italy [70, 71, 84, 85]. Even if testing capacity for millions of patients were theoretically available early on, but initial sensitivity of antigen and PCR tests proved lacking, imaging was an irreplaceable tool to increase diagnostic certainty for suspected cases, creating large amounts of imaging data that might have formed the basis for analysis and model development [14, 38, 86, 87].

Instead of coordinated issuing of recommendations on the use of radiology in respiratory pandemics or providing early radiological descriptions and typical images to support radiologists in clinical practice by a single organization, various national and international societies independently developed inhomogeneous guidelines as a response to missing standardization and data sharing [72–74, 77]. Dissemination of information through radiological publications took months to commence (e.g., H1N1 or MERS, cf. Fig. 2) and typically reported single-center retrospective data [4, 88–90]. This has caused multiple nations to request radiological prevalence- and resource-adapted advice from the WHO only recently during COVID-19 [67, 74].

In terms of data collection, case reporting forms were created each time after an outbreak was already underway, and in contrast to clinical information, radiological data were almost entirely omitted. This reactionary approach targets verbal case descriptions and does not provide the data basis or infrastructure to rapidly gather quantitative information together with two- or three-dimensional imaging data required for training of AI models that could support diagnosis and treatment decisions [28, 44, 45, 58, 64, 91]. In case of MERS, no successful data collection efforts are known to date, although it is considered to be a priority pathogen (see Table 1), and complete information on previous cases would be of utmost importance in case of rapidly increasing prevalence [58–60]. As data collection is a time-consuming task, it is not surprising that initiatives repeatedly lack success when they impose additional bureaucratic tasks on clinicians during an outbreak, instead of setting up automated data transmission and curation pathways prior to the next pandemic.

**Table 1:** WHO list of Blueprint priority diseases as of August 2024

• COVID-19
• Crimean-Congo hemorrhagic fever
• Ebola virus disease and Marburg virus disease
• Lassa fever
• Middle East respiratory syndrome coronavirus (MERS-CoV) and Severe Acute Respiratory Syndrome (SARS)
• Nipah and henipaviral diseases
• Rift Valley fever
• Zika
• “Disease X”: a currently unknown human pathogen, for which preparedness is also urgently needed

Not only the multinational collection of imaging data, but consequently also the processing and dissemination have been widely uncoordinated. As a reaction to the lack of internationally coordinated successful efforts in this direction, the RICORD or STOIC projects, among others, were launched, but it took at least 1 year until their results were publicly accessible [81, 82]. As early as 2004, a lack of legal framework for data sharing has been noted, but questions about ownership or publication rights remain unanswered to some extent to this day, greatly hindering future pandemic preparedness [92].

In summary, we identified the following key issues for pandemic preparedness [93–95]:

- Missing international structures and capacities for real-time radiological and clinical data collection, curation and distribution in close collaboration with experts from the field during surveillance and periods of present pandemic disease.
- Lack of a single, neutral, international institution explicitly designated for the abovementioned task of radiological and clinical data collection.
- Data sharing, collection and distribution hindered by a missing framework of legal and political regulations.
- No structures for swift AI algorithm development, validation, and deployment available.
- The need for a shift from pandemic response to pandemic preparedness.

Based on the findings of previous studies [9, 12, 93], combined with the shortcomings identified in this work, we have compiled the following recommendations in Table 2.

**Table 2:** Priorities, requirements, and actions for pandemic preparedness

1—Collection and curation of representative data during surveillance and outbreaks
• Create capacity for real-time continual structured routine data collection and dissemination while minimizing the additional burden for physicians and radiologists as part of continual surveillance, and to expand collection and dissemination capacity rapidly during an outbreak.
• Establish expert panels to guide collection, curation and annotation, define minimum standards for data collection taking available resources into account.
• Define frameworks for governance, management, and legal compliance, ensuring data privacy, security, intellectual property, and risk.
2—Development and validation of reliable AI algorithms
• Disseminate comparable, diverse, fair, quality-controlled, and globally representative data to the global scientific AI community to enable distributed method development, engaging a diverse and geographically distributed group of researchers.
• Make mechanisms for comparative evaluation and benchmarking of AI algorithms available to ensure validation of algorithms that benefit the diverse global patient population and a heterogeneous healthcare landscape.
• Implement infrastructure for the deployment of algorithms for (a) early detection of new outbreaks in routinely acquired data, and (b) for the training and deployment of algorithms for treatment guidance based on initial observational data during an outbreak.
• Develop AI models to detect newly emerging diseases in large-scale global routine data with high sensitivity to enable timely identification of possible outbreaks.
• Develop AI technology to train models for the diagnosis and treatment guidance based on minimal in-homogeneous initial observational data representing a wide patient population, coping with heterogeneous care during the early stage of a pandemic.
3—Delivery of AI during surveillance and outbreaks
• Create mechanisms to share methods and algorithms for wide use and continued development in research and comparison studies. Support translation to clinical use including its regulatory prerequisites.
• Provide algorithms and processing capability for continual worldwide surveillance to detect emerging outbreaks coping with the diversity and continual change of imaging technology, patient populations, diseases, and healthcare systems.
• Provide systems for the rapid global deployment of AI algorithms for diagnosis, prognosis, and treatment decision support during a pandemic outbreak.
• Rapidly create teaching materials for radiologists created and edited by an established expert panel and mechanisms to compile, assess, and interpret data collected globally.
4—Implementing a global disease phenotype observatory
• Establish the structure and funding of an observatory to implement these actions governed by a single existing neutral multinational organization, and advised by an international expert panel to address the above-listed requirements.
• Develop a clear cooperation with national and multinational radiological- and public health organizations.
• Recruit clinical centers globally to participate in the observatory program to ensure wide applicability and benefits of the observatory
• Engage the clinical and the AI research community to use the observatory for research and development and to contribute their findings to the observatory for implementation in a productive phase, and for down-stream research across communities.
• Define KPIs linked to clinical impact for infrastructure, data diversity, collection- and dissemination capacity, algorithmic benchmarks, and availability of curated data within 2 weeks after a suspected outbreak.

## Conclusions

While imaging played a critical role in all analyzed respiratory pandemics of the 21st century, data collection and analysis during emerging respiratory outbreaks were repeatedly hampered due to missing international structures, capacities, and legal frameworks. This is critical since AI technology would have been able to support and accelerate detection, diagnosis, and treatment guidance. However, its availability was delayed due to a lack of data until late in the outbreak, and its clinical applicability was hindered by missing international frameworks. Evidence shows that AI, together with routinely collected data, bears substantial potential in helping us detect and react to future outbreaks. Until a coordinated global detection and response system is up and running the inevitable question remains—ready for disease X?

## Abbreviations

(RT-)PCR: (Reverse transcription-) polymerase chain reaction
AI: Artificial intelligence
CDC: Centers for Disease Control and Prevention
COVID-19: Coronavirus disease 2019
CT: Computed tomography
DGE: Mexican General Directorate of Epidemiology
H1N1: Influenza-A-virus H1N1
ICU: Intensive care unit
IHR: International Health Regulations
MERS: Middle East Respiratory Syndrome
MRI: Magnetic resonance imaging
RICORD: RSNA International COVID-19 Open Radiology Database
RSNA: Radiological Society of North America
SARS: Severe acute respiratory syndrome
WHO: World Health Organization
